# Why balance matters more than totals: a candidate structural model of non-compensable wellbeing, duratva, and Buddhist contemplative practice

**DOI:** 10.3389/fpsyg.2026.1902817

**Published:** 2026-08-31

**Authors:** Swapan Samanta

**Affiliations:** Indoriv Clinical Research Centre, Kolkata, India

**Keywords:** autonomy, Buddhist contemplative practice, dūratva, equilibrium geometry, flourishing, jivanmukti, non-compensability, PERMA

## Abstract

**Problem:**

A persistent observation troubles both positive psychology and contemplative science: people suffer in the midst of success. The wealthy report emptiness; the deeply loved report captivity; the lucid report paralysis; the renunciate remains seized by the identity of renouncing. Existing wellbeing frameworks—utility theory, composite indices, PERMA—explain this poorly because they share a hidden structural assumption: compensability. If goods are compensable, a high total score should predict wellbeing regardless of balance. It does not.

**Gap:**

Contemporary wellbeing frameworks are multidimensional but remain compensable: they allow surplus on one dimension to offset deficit on another. Buddhist contemplative science documents what practice produces, but neither field has identified the structural mechanism connecting practice to flourishing. This paper proposes that the missing structure is non-compensability, and that formalizing it yields both a theory of flourishing and a candidate structural account of liberation-in-life.

**Model:**

The Equilibrium Geometry of Flourishing locates a life in a four-dimensional state space with coordinates Dhana (sustaining sufficiency), Samsara (relational reciprocity), Vidya (critical consciousness), and Vairagya (autonomy-as-non-seizure). The equilibrium surface L represents balanced flourishing; duratva—the distance from this surface—is the primary scalar measure of avoidable suffering. Non-compensability is the structural axiom: as any coordinate approaches its lower threshold, duratva grows steeply toward its maximal finite value regardless of surplus on the others. A Burden Law, derived from this geometry, shows why acquisition without proportional Vairagya increases duratva rather than reducing it.

**Predictions:**

The model yields eight falsifiable hypotheses (H1–H8). H1: balance outpredicts totals in wellbeing surveys. H2: unmatched single-axis gains reduce long-run flourishing. H3: high Vidya with low Vairagya predicts a specific distress profile. H4: recovery rate after perturbation scales with Vairagya. H5: rebalancing outperforms single-axis intensification above threshold. H6: raters blind to the model sort cross-tradition symbols onto the four dimensions above chance. H7: Buddhist practitioners addressing at least three dimensions show steeper Liberation Coefficient growth. H8: PERMA profiles cluster dimensionally by practice type.

**Implications:**

The geometry recovers the tradition’s own paradigm-case verdicts (Janaka near the surface; the lottery winner far from it), identifies jivanmukti with stable equilibrium maintenance under acquisition, and provides a structural foundation for why all four canonical Buddhist paths are simultaneously required. The result is not a doctrinal prescription but a geometric consequence of non-compensability.

## 1 Introduction

*The acquisition was real. The arrival was not.*

*artham labdhvapi duhkhartha kasmad bhavati manavah / na hi sthanam kintu samyak-sthitih sukhasya karanam // Why does a person, even having gained the thing, remain stricken with sorrow? Not the position, but the right standing within it, is the cause of ease.*

Consider four people.

The first has accumulated more material wealth than any of his ancestors could have imagined. His house is quiet and large. His calendar is a monument to his achievements. And yet at fifty-three, he sits across from me—I am a cardiologist, and his heart is technically sound—and tells me he feels, in a word he uses carefully, *empty*. Not sad. Not angry. Empty. As though the filling of the vessel proved the vessel was not the problem.

The second is a woman whose relational life overflows in every direction. She is loved. Her family depends on her, her friends call her first, her colleagues trust her without question. She cannot remember the last time she said no. By any additive measure of social connection she is exceptionally rich. She has not slept through the night in 4 years.

The third is a man of exceptional critical intelligence. He can see the system that holds him: how his desires were installed by a social field that needed him to want exactly what he wants. He sees it all. He cannot, despite this seeing, change a single significant thing. His clarity has become its own captivity.

The fourth has renounced. She lives simply, meditates 4 h each day, and is recognized as someone who has achieved spiritual distance from the world. When I ask whether she is free, she pauses. Then: “*I am free from the world. I am not free from the person who is free from the world.*”

These four people share one condition: they are suffering in the presence of surplus. The additive models that dominate both positive psychology and popular wisdom cannot account for this. The billionaire’s total score is not low—it is high, dragged upward by one enormous coordinate. The reports are of imbalance at high totals, and averaging is precisely the operation that erases imbalance.

The present paper develops the structural geometry. Subsequent papers in the program address the neurophysiological mechanisms through which contemplative practice alters position within this geometry, the embodied and somatic pathways through which the dimensions are cultivated, the implications for clinical and educational intervention design, and the cross-cultural and ethical questions raised by Buddhist-positive psychology integration.

### 1.1 The research gap: multidimensional but still compensable

Contemporary wellbeing research has responded to the insufficiency of purely hedonic accounts by proliferating dimensions. Seligman’s PERMA framework (Seligman, 2011) identifies positive emotion, engagement, relationships, meaning, and accomplishment. The capability approach (Sen, 1999; Nussbaum, 2011) enumerates the functionings and freedoms that constitute a good human life. Self-determination theory (Deci and Ryan, 2000) identifies autonomy, competence, and relatedness as basic psychological needs. Each is a genuine advance. Each, however, retains the assumption that made the single-dimension model inadequate: compensability. A high score on one dimension offsets a low score on another. The sum, the average, or the composite determines the verdict.

The paper’s novelty is the formalization of non-compensability as a structural axiom. This is not a new empirical claim—the wellbeing literature has documented compensability failures for decades, from the Easterlin paradox (Easterlin, 1974) to hedonic adaptation (Brickman and Campbell, 1971; Brickman et al., 1978) to income-wellbeing flattening (Kahneman and Deaton, 2010), to the paradox of declining female happiness (Stevenson and Wolfers, 2009). It is a structural claim: these failures are not noise in the data but consequences of the correct geometry. Once non-compensability is made axiomatic, a measurement framework that has always been lurking in the clinical and philosophical literature becomes explicit, falsifiable, and applicable.

Buddhist contemplative science contributes a parallel insight: the four canonical paths—karma yoga, metta-bhavana, vipassana, and shamatha—are presented in the tradition not as alternatives but as simultaneous requirements. The tradition does not explain why. The equilibrium geometry does: each path primarily addresses one non-compensable dimension, and no single path can substitute for the others precisely because the dimensions are not compensable. This is the structural bridge between Buddhist practice and positive psychology that the Research Topic seeks.

### 1.2 Methods

This study employs a theoretical, integrative, and model-building methodology. Rather than collecting new empirical data, it develops a formal framework through the synthesis of Buddhist philosophical concepts, contemporary theories of psychological flourishing, and principles drawn from mathematical modeling and systems thinking.

The development of the model proceeded in three stages. First, recurrent themes concerning human flourishing were identified across Buddhist sources and contemporary wellbeing research. Particular attention was given to phenomena that conventional additive models struggle to explain, including persistent suffering despite material success, relational fulfillment accompanied by existential dissatisfaction, and other forms of structural imbalance. These observations motivated the search for a framework capable of representing flourishing as a multidimensional equilibrium rather than a collection of independent gains.

Second, four candidate dimensions were selected based on their theoretical recurrence and functional distinctiveness: sustaining sufficiency (Dhana), relational reciprocity (Samsara), critical consciousness (Vidya), and autonomy-as-non-seizure (Vairagya). These dimensions were then formalized within a geometric state-space representation, allowing the development of the dūratva metric and the derivation of associated principles such as non-compensability and the Burden Law.

Third, the framework was subjected to internal consistency analysis and theoretical stress testing. Mathematical formulations were examined for coherence with the underlying conceptual claims, and explicit falsification conditions were developed. Rather than presenting the framework as an established account of flourishing, the paper treats it as a candidate theory whose value depends upon its ability to generate precise predictions and survive empirical evaluation.

Accordingly, the study occupies a position between philosophical interpretation and empirical science. Its immediate objective is explanatory and theoretical; its longer-term objective is to provide a structured foundation for instrument development, longitudinal testing, intervention research, and cross-cultural investigation.

## 2 The four dimensions of flourishing

*Functional definitions, measurable indicators, and contemplative antidotes*

**FIGURE 1:**
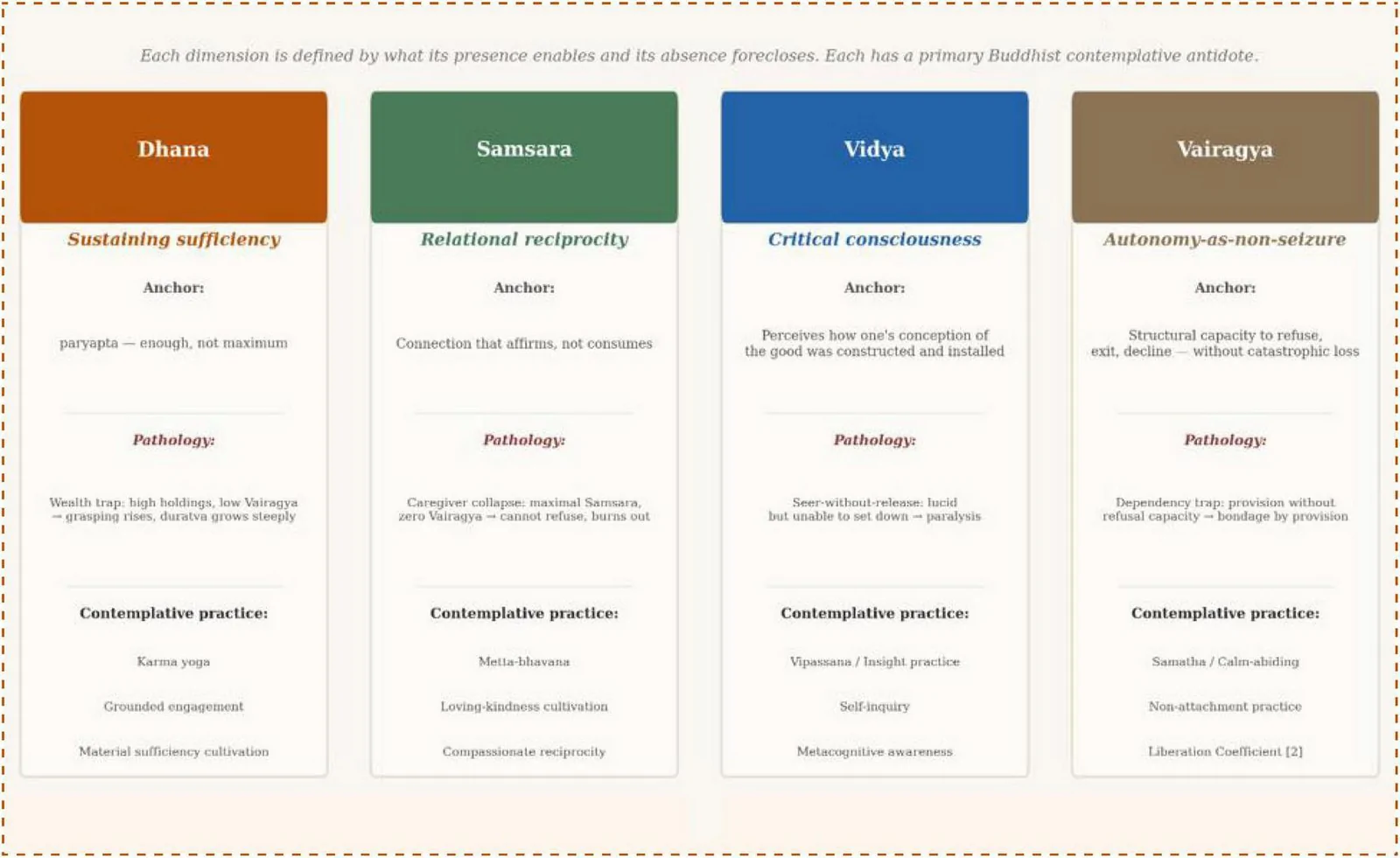
The four irreducible dimensions of flourishing. Each dimension is defined by what its presence enables and its absence forecloses, with its canonical pathology and primary contemplative antidote.

**FIGURE 2:**
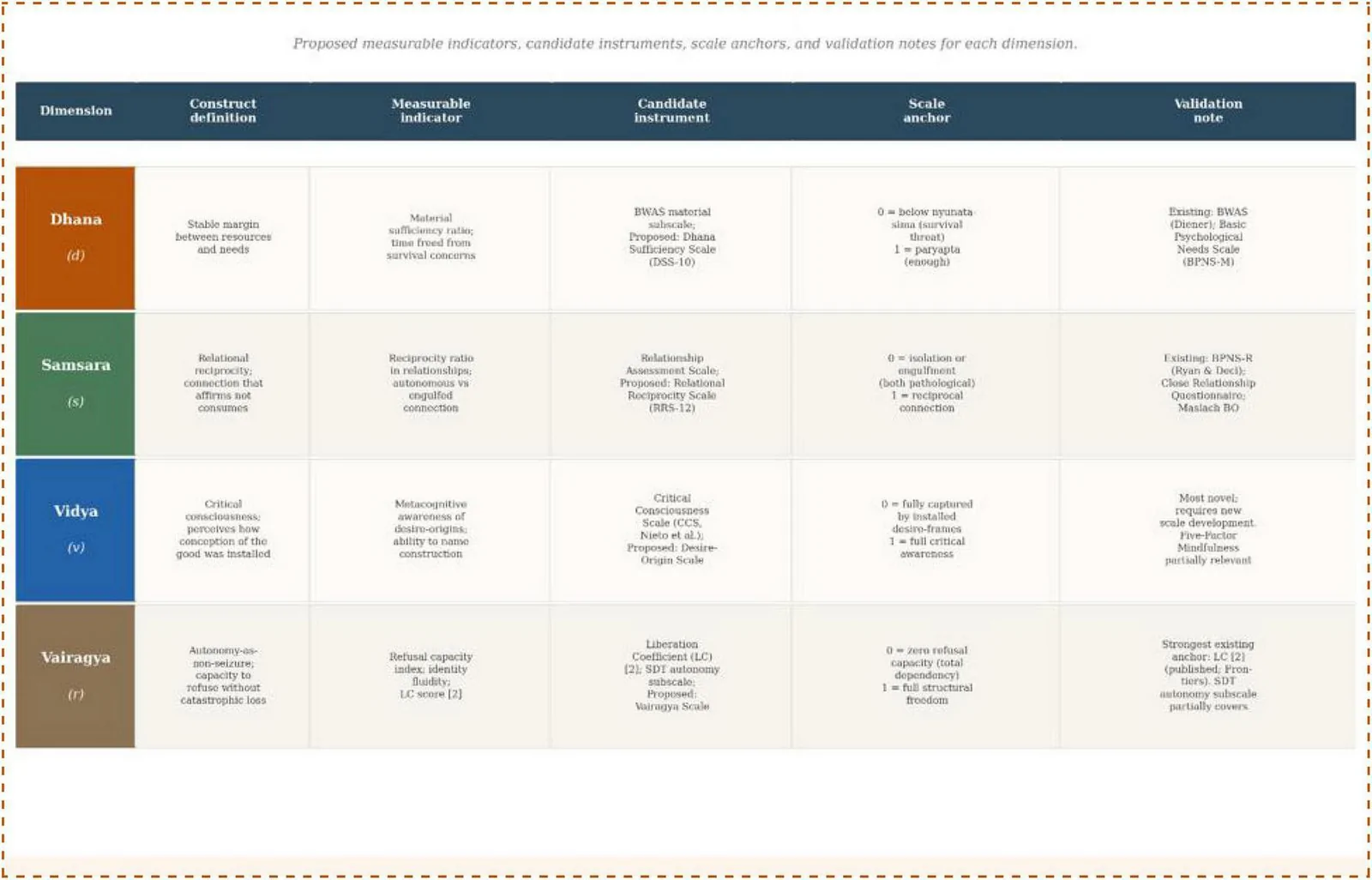
Operationalization of the four dimensions. Proposed measurable indicators, candidate instruments, scale anchors, and validation notes. The Vairagya dimension has the strongest existing measurement anchor (LC; Samanta, 2026); Vidya requires the most substantial new instrument development.

The four dimensions are defined functionally—by what their presence enables and their absence forecloses—so that each is in principle operationalizable. Figure 1 shows the complete dimensional architecture; Figure 2 maps each dimension to measurable indicators and candidate instruments (see also Supplementary Appendix C).

### 2.1 Dhana—sustaining sufficiency

Dhana (Sanskrit: resource, material holding) is sustaining sufficiency: the stable margin between resources and needs that frees attention from survival preoccupation. Two clarifications are required. First, Dhana is objective-resource-grounded rather than purely subjective: below the lower threshold (*nyunatā-sīmā*), genuine material deprivation is present and the model says so plainly—this is not a framework that romanticizes poverty. Second, Dhana is not wealth maximization but a threshold good: helpful below *paryāpta* (enough), and either inert or burden-generating above it. The distinction between objective resources and subjective sufficiency is operationally important: two people with identical incomes may differ sharply in Dhana if their needs differ, and the scale anchor is calibrated to *paryāpta* rather than to a cultural maximum.

### 2.2 Relational reciprocity (S_R)—Sanskrit gloss: samsara

Samsara is used here in its etymological sense—the balanced flowing-together, the circular reciprocity—rather than in its later metaphysical usage as the cycle of conditioned existence. This usage requires explicit justification against Buddhist studies reviewers: the term is chosen precisely because the etymological sense captures relational balance, and the paper uses it as a technical term with that restricted meaning throughout. To make this technical status unmistakable, the main text hereafter employs the explicit label Relational Reciprocity (S_R), with the Sanskrit term retained as a gloss; readers in Buddhist studies may thus treat S_R as the operative construct and samsara as its etymological ancestry rather than its doctrinal content. Readers in the Buddhist studies tradition are invited to read it as *sam-sara* (balanced flow) rather than as the metaphysical *samsara* (the cycle of rebirth). Its pathologies are two-directional: isolation below threshold, and the dissolving over-connection that erases autonomy above it.

### 2.3 Vidya—critical consciousness

Vidya (Sanskrit: seeing, knowledge) is the most theoretically novel dimension and deserves extended treatment. It is not credentialed knowledge, not intelligence, and not mindfulness in the contemporary clinical sense. It is the capacity to perceive how one’s own conception of the good has been constructed and installed by the social field: to see that the desires one experiences as natural and spontaneous were in significant part given, shaped, and reinforced by structures that benefited from one’s wanting exactly this.

Vidya comprises two distinguishable sub-capacities, and the distinction matters both for measurement and for the independence argument of Section 4. *Vidya-P* (perceptual) is the meta-cognitive capacity to perceive that one’s desires were socially constructed—to see the installation. *Vidya-E* (evaluative) is the capacity to critically assess whether those desires, once seen as constructed, are worth endorsing—to judge the installed. The two dissociate in both directions: a person may see the construction with clinical precision yet possess no framework for evaluating what to do with the seeing (V-P without V-E), and a person may hold refined evaluative standards while remaining blind to the installed character of the standards themselves (V-E without V-P). The composite coordinate v is therefore modeled as requiring both sub-capacities; the proposed scale items in Supplementary Appendix C are tagged [P] or [E] accordingly, and the separability of the two sub-factors is an explicit, pre-registered target of the measurement program (Supplementary Appendix D, Study 2).

For readers working within existing psychological frameworks, a brief mapping is useful. Vidya-P corresponds broadly to meta-cognitive awareness of socially constructed preferences—the capacity addressed, in its political form, by the Critical Consciousness Scale (Diemer et al., 2017); the present construct extends that capacity to existential and contemplative contexts. Vidya-E corresponds to what Frankfurt called second-order volition and what deliberative autonomy theorists call reflective endorsement: not merely observing a desire but evaluating whether to identify with it. Vairagya extends SDT’s autonomy construct by adding the structural dimension of *exit capacity*: SDT autonomy concerns the quality of motivation (intrinsic vs. extrinsic), (Ryan and Deci, 2017), while Vairagya concerns whether the configuration of one’s life—roles, identities, attachments—can be held and released without survival threat, relational rupture, or self-deception. A person can be intrinsically motivated within a seized position; Vairagya addresses the seizure itself.

This dimension has no precise correlate in PERMA, SDT, or the capability approach, which is its chief claim to originality. The closest existing construct is Bourdieu’s *doxa* (Bourdieu, 1990)—the pre-reflective acceptance of the social world as given—and Vidya is the capacity that makes doxa visible to itself. Freire’s *conscientizacao* (Freire, 1970) is the political-pedagogical form of the same capacity. In the contemplative tradition, vipassana’s sustained observation of the arising and passing of mental phenomena serves the same structural function: it makes the fabricated nature of the current orientation visible, which is the precondition for the capacity to refrain from re-fabricating it.

The Vidya-Vairagya dissociation (section 4) is the paper’s key structural argument. Seeing clearly is not the same as being able to set down what one sees. This dissociation—the seer who cannot act on perception, the free person who exits the wrong things—is what requires Vairagya to be an independent axis rather than a consequence of insight.

### 2.4 Vairagya—autonomy-as-non-seizure

Four common misreadings of Vairagya must be distinguished. Vairagya is not (i) detachment in the sense of affective blunting or emotional suppression; (ii) avoidance—the person who cannot engage is not high in Vairagya, they are low in all four dimensions; (iii) voluntary poverty—the destitute person forced below the Dhana threshold has low Vairagya precisely because they cannot exit their deprivation; (iv) spiritual indifference—the teacher’s instruction to act without clinging to outcomes (*nishkama karma*) is an account of high Vairagya in the presence of full engagement, not absence of engagement.

Vairagya is the structural capacity to refuse, exit, or decline—without catastrophic loss. Its operational signature is the ability to exit a role, relationship, or resource configuration voluntarily when it no longer serves one’s deepest values, without this exit threatening survival (Dhana), destroying relational bonds (Samsara), or requiring self-deception (Vidya). This is precisely what the Liberation Coefficient (LC) of the companion framework (Samanta, 2026) measures: position-fixation approaching zero means the identity is no longer seized by what it holds. The present paper provides the four-axis context in which LC acquires its full meaning.

### 2.5 Why exactly four? Selection criteria and the subsumption of rival dimensions

The selection of exactly these four dimensions follows four explicit criteria, applied jointly. (i) *Functional irreducibility*: each dimension must be dissociable from the others in clinically or contemplatively recognizable presentations—the dissociation arguments of Section 4 are the paradigm. (ii) *Dual-threshold structure*: each dimension must exhibit both a deprivation floor (nyunatā-sīmā) and a sufficiency anchor (paryāpta), below and above which its behavior changes qualitatively. (iii) *Contemplative addressability*: a canonical contemplative path must exist whose primary action is on that dimension (section 8). (iv) *Non-compensability with the remaining dimensions*: collapse on the candidate axis must not be repairable by surplus elsewhere.

These criteria explain why constructs prominent in existing frameworks are subsumed rather than added. *Meaning and purpose* are treated not as a fifth axis but as the phenomenology of proximity to the equilibrium surface: the felt sense of meaning is what low dūratva is like from within, which is why meaning collapses under imbalance even when its supposed ingredients are individually abundant. *Physical vitality* is a component of the Dhana resource base—the body is the first resource, and somatic depletion registers as Dhana shortfall against a paryāpta calibrated to functional need. *Efficacy-based agency* is distributed across two axes: perceiving one’s options is Vidya, and the structural capacity to act on or exit them is Vairagya; treating agency as a single further axis would re-merge precisely the capacities whose dissociation section 4 establishes.

The framework’s commitment is to at-least-these-four, with exactly-four as the working structural claim. The claim is defeasible in a specified way: a well-developed tradition or dataset encoding a genuinely different cardinality—not a relabeling—would require extension of the model (Limitation 4; the rater-recovery test H6 is the designed empirical avenue for this challenge). Three frameworks serve as explicit alternative-cardinality benchmarks: SDT’s three basic psychological needs (Ryan and Deci, 2017) (autonomy, competence, relatedness) represent a substructure of the present four; Wallace and Shapiro’s (2006) four-domain model (conative, attentive, cognitive, affective) represents a same-cardinality but structurally distinct competitor; and the Capabilities Approach’s open-ended list represents a potential superstructure. The measurement-invariance program (Study 3, Supplementary Appendix D) and the rater-recovery test (H6) are designed precisely to arbitrate between the present four-axis proposal and these alternatives—not to pre-empt them.

## 3 The principle of non-compensability

*Structural axiom, cross-domain analogs, and geometric expression*

Proposition 1 (structural axiom): flourishing is non-compensable. A deficit on any dimension cannot be repaired by surplus on another. This is the paper’s central committed claim, and it is the right place to ask: what does non-compensability mean precisely, and why should we accept it as a structural feature rather than an empirical one?

Non-compensability appears in multiple domains as a discovered structural property rather than a doctrinal preference. In medicine: a patient’s hemoglobin level cannot be offset by excellent renal function—the system has a minimum-requirement structure, not a weighted-sum structure. In ecology: the minimum law of Liebig (von Liebig, 1840) states that plant growth is limited by the nutrient in shortest supply, not the nutrient average—a classic non-compensable system. In engineering: structural safety under combined loading follows a product-coupled failure criterion (von Mises stress), not an additive one—components at their individual limits produce failure at lower combined loads than additive models predict. In each case, balance across dimensions matters independently of the total. The present paper proposes that flourishing has the same structural property. The epistemic status of these analog must be stated exactly: they are motivating precedents. They establish that non-compensable structures are common and respectable in nature; they do not establish that flourishing is one. Whether it is, is what the hypotheses of section 11 (H1–H8) are constructed to decide.

**FIGURE 3:**
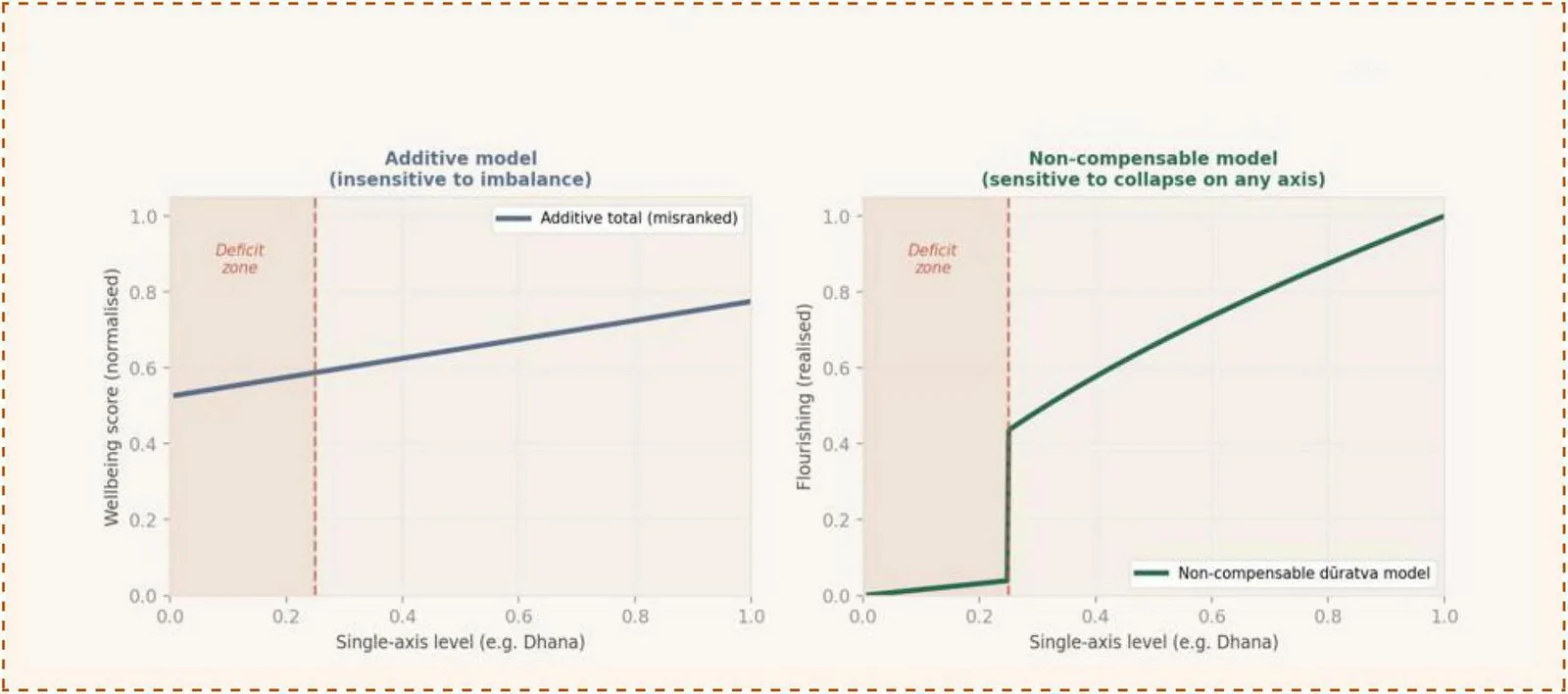
The compensability problem. Additive model (left): insensitive to imbalance; barely registers collapse on one axis. Non-compensable geometry (right): collapse on any axis drives flourishing toward zero regardless of other scores. The shaded band marks the deficit zone below nyunatā-sīmā.

The rectangle analogy remains the most accessible intuition: the area of a rectangle is its length times its width; no elongation of one side compensates for the collapse of the other. Figure 3 contrasts the two model classes directly. The four dimensions of flourishing behave likewise. As any coordinate approaches its lower threshold, the realized flourishing approaches zero irrespective of surplus elsewhere.

## 4 The independence of Vidya and Vairagya

*The argument that converts a wellbeing geometry into a liberation geometry*

This section establishes one narrow claim on which everything in section 7 depends: Vairagya is a genuinely independent coordinate—not a consequence of Vidya, not a manner of holding the other dimensions lightly. If this independence fails, the model collapses to three dimensions and becomes one more multidimensional wellbeing theory.

**FIGURE 4:**
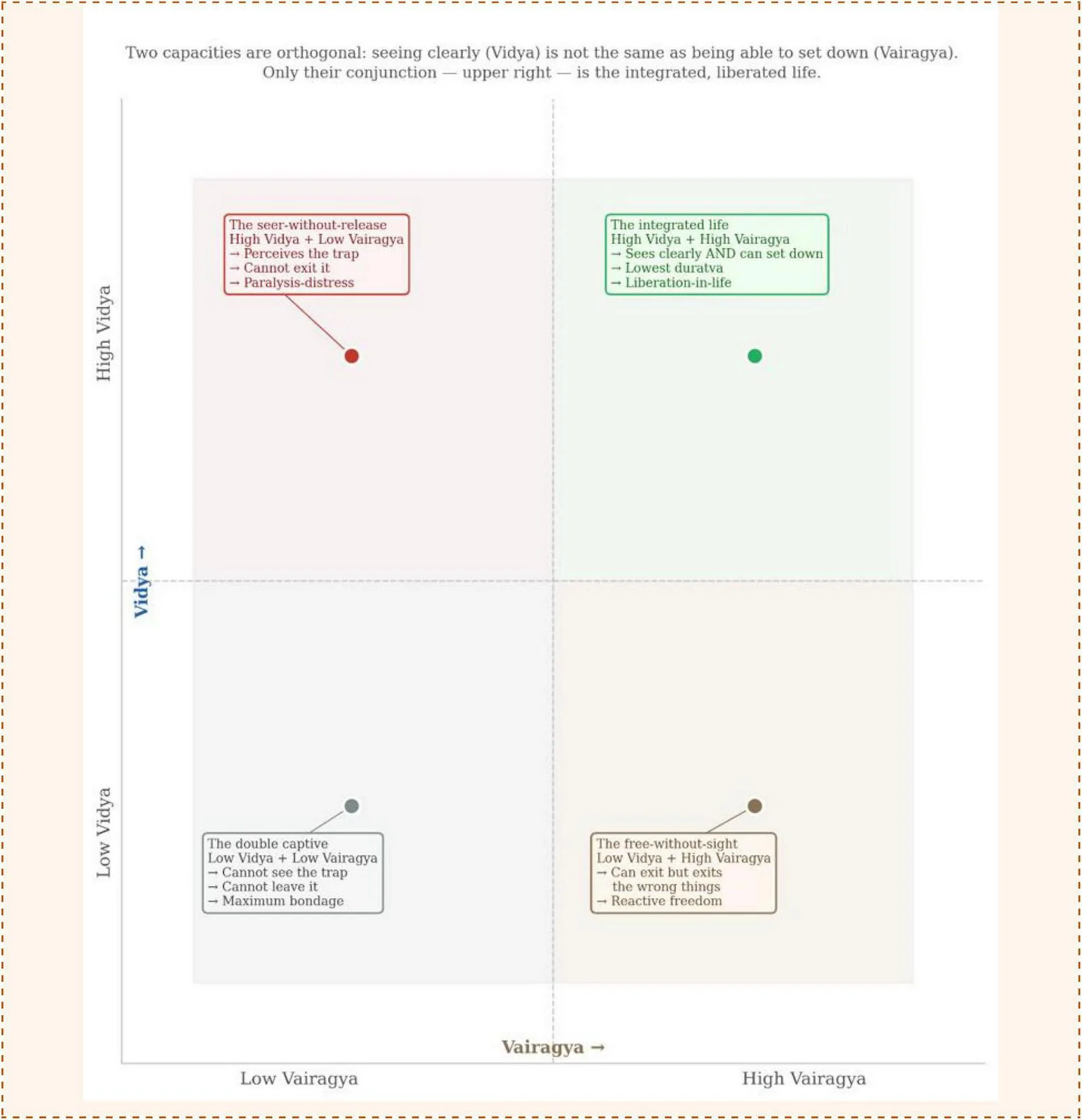
The Vidya-Vairagya independence matrix. Seeing clearly (Vidya) and being able to set down (Vairagya) are orthogonal capacities. Only their conjunction (upper right) is the integrated life. Each quadrant corresponds to a clinically and contemplatively recognizable presentation.

The argument is a dissociation in the clinical and logical sense. The *seer-without-release* (high Vidya, low Vairagya) perceives with precision the system that holds them and remains in it—because perception is not the same capacity as structural exit. The *free-without-sight* (high Vairagya, low Vidya) exits readily and exits the wrong things, because freedom without perception is reactive rather than navigated. These two figures occupy opposite quadrants of a single plane (Figure 4). A single axis cannot place a person in opposite quadrants. The capacities are orthogonal. The bifurcation of Vidya into perceptual and evaluative sub-capacities (section 2.3) sharpens rather than weakens this argument: the seer-without-release may possess V-P alone, or V-P together with V-E; in either configuration the missing capacity is structural exit, which neither sub-capacity of seeing supplies.

*Remove Vairagya as a coordinate and you are left with a theory of wellbeing. The fourth axis is what makes the geometry a theory of liberation rather than of comfort.*

## 5 The equilibrium geometry and the dūratva measure

*State space, equilibrium surface, committed form of f, and computable measure*

### 5.1 State space and equilibrium surface

Represent a life as a point E = (d, s, v, r) in the unit four-cube [0,1]^4^, where d, s, v, r are the normalized levels of Dhana, Samsara, Vidya, and Vairagya respectively. Not every point in this space is a flourishing one. The equilibrium surface L is the locus of balanced states:

*Equilibrium surface (Definition)* L = { E : f(E) = 0 }

where f is a balance constraint function whose committed form is specified in §5.2. Two thresholds shape each axis: *nyunatā-sīmā* (n_dim), the lower bound below which genuine deficit is present; and *paryāpta* (p_dim), the sufficiency anchor above which further gain yields no increase in flourishing and may generate burden. These thresholds are empirical parameters to be estimated; their existence as a structural feature is the model’s commitment.

### 5.2 Committed form of f and the dūratva metric

Prior treatments left f as an open empirical question. To generate predictions that follow from the mathematics rather than from intuition, this paper commits to a candidate form. Let each axis carry a normalized shortfall below its sufficiency anchor:

*Shortfall on axis i (normalized)* q_i = max(0, p_i - x_i)/p_i where x_i is the observed score

The grasping factor g is derived—not a fifth coordinate—and encodes how far Vairagya falls short of what is held:

*Grasping (derived quantity)* g = (d + s + v)^2^/(r + ε) where ε = 0.05 prevents division by zero

Two grasping quantities appear in the committed form, and their difference is functional, not accidental. The global factor g = H^2^/(r + ε) is quadratic in holdings: the total burden of holding compounds with the total held, so the multiplier that scales the whole distance grows with the square of aggregate holdings. The axis-specific pressure g_r = H/(r + ε) is linear in holdings: the pressure exerted on the release axis is proportional to what is held. The quadratic form governs the global multiplier; the linear form governs the local coupling on the Vairagya shortfall. The constant ε is a regularization parameter preventing singularity at r = 0; the value 0.05 is a working choice, not a theoretical commitment—it should be treated as an empirical parameter, and model outputs must be checked for sensitivity to it wherever r is very small (Supplementary Appendix B reports this sensitivity analysis for the Caregiver case, r = 0.10).

Dūratva is then defined as the product-coupled distance from the equilibrium surface, incorporating the grasping multiplier:

*Dūratva measure d(E)—committed form* d(E) = √[q_d^2^ + q_s^2^ + q_v^2^ + q_r^2^ (1 + g_r)^2^] × (1 + αg) where g_r = H/(r + ε)

The structural choice that encodes non-compensability is the product-coupled form: near a collapse on any axis (q_i → 1), the distance grows steeply toward its maximal finite value regardless of surplus elsewhere, so no compensation is possible. Because each shortfall is bounded (q_i ≤ 1), dūratva does not diverge in the strict mathematical sense; it grows toward a large finite bound—large enough, as the worked examples of Supplementary Appendix B show, to dominate every other contribution. A terminological clarification is also required: d(E) is a scalar field on the state space—the distance of a life-state from the equilibrium surface—not a metric in the strict mathematical sense, since metric axioms (symmetry, the triangle inequality) are defined for pairwise distances between points and do not apply to a point-to-surface distance. The text therefore refers to d(E) as the dūratva measure. The parameters—threshold values p_i, n_i, and the grasping coefficient—are empirical and must be estimated from data; what the paper commits to is the qualitative structure of the form, not specific parameter values.

## 6 The burden law

*Formal statement and derivation from non-compensability*

The Burden Law is the dynamic consequence of the static geometry. Because balance—not magnitude—defines the equilibrium surface, any gain on a single axis raises the level required on the others to remain balanced. An unmatched gain therefore moves a life *away* from the surface. This is not an empirical generalization; it follows directly from the committed form of f.

Formally: let H = (d + s + v) be the aggregate of holding-axis scores at time t. The Burden Law states the marginal dūratva cost of a gain dH:

*Burden Law—differential form (full chain rule; S denotes the inner square root, M = 1 + αg)* ∂d(E)/∂H = (1/(r+ε)) × [2αH⋅S + M⋅q_r^2^(1+g_r)/S] > 0 off the surface; = 0 on the surface

**FIGURE 5:**
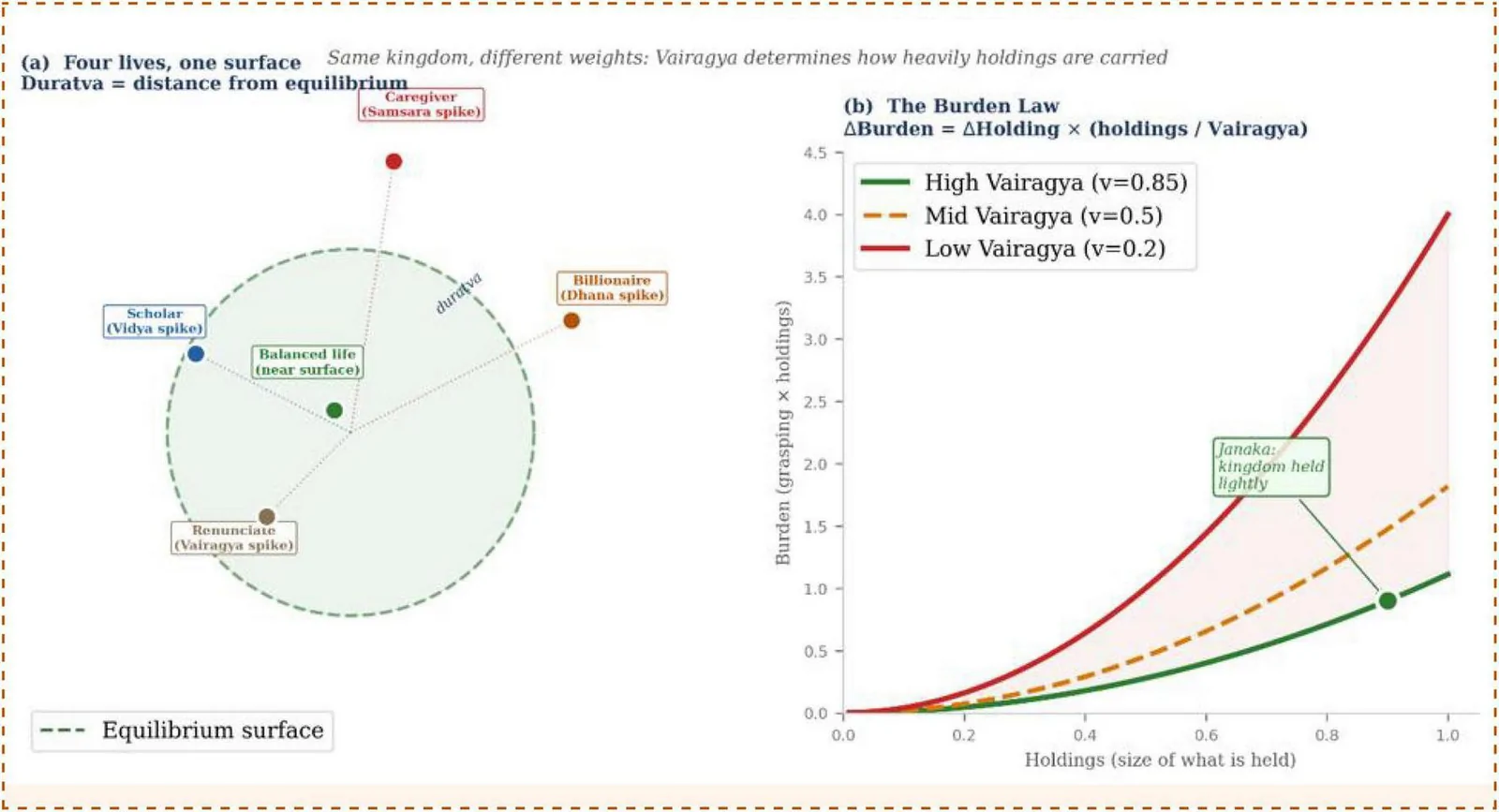
**(a)** Equilibrium geometry: the four paradigm lives and their dūratva. **(b)** The Burden Law: same kingdom, different weights. At high Vairagya (green) the Janaka profile holds vast holdings with near-zero burden increase. At low Vairagya (red) even modest holdings are heavy.

The key structural result is twofold. First, the derivative is strictly positive for every life off the equilibrium surface: wherever any shortfall remains, acquisition increases dūratva. Second, the derivative vanishes identically on the surface: when every shortfall is zero, acquisition adds no dūratva at all. Here is the precise sense in which Janaka holds a kingdom weightlessly, and it is the exact statement that replaces the looser formulation ‘the derivative approaches zero at r = H’—the Janaka condition is residence on the equilibrium surface with Vairagya at the level of the holdings, not the algebraic identity r = H (which is not generally attainable within [0,1]^4^ when H > 1). Off the surface, the steepness of the burden increase is set by Vairagya through the 1/(r+ε) factor and the g_r term: at high r, the same acquisition barely increases dūratva; at low r, the increase is steep. Figure 5 shows both the paradigm-life geometry and the resulting burden curves.

Proposition 2 (Burden Law): Unmatched acquisition increases dūratva. The rate of increase is inversely proportional to Vairagya. Only on the equilibrium surface—where every shortfall is zero—does acquisition leave dūratva unchanged.

## 7 Bondage and liberation

*A candidate structural model of jivanmukti*

*ekam bharam parityajya dvau bharau yo grihitavan / muktir iti sa manyeta dvi-gunam bandhanam tu tat // He who, setting down one burden, takes up two—and calls it freedom: that is bondage doubled.*

### 7.1 Bondage as persistent dūratva

Proposition 3 (candidate structural model): bondage is persistent dūratva. The word *persistent* carries the full epistemic weight: a momentary high dūratva is ordinary life turbulence, not bondage. What the tradition calls *bandha* is the settled, self-maintaining configuration far from the surface—a state that has become stable enough to resist return.

This claim is defensible against the addiction science literature. Robinson and Berridge’s incentive-salience model (Robinson and Berridge, 1993) describes addiction as a stable attractor state in the wanting system, dissociated from hedonic experience—wanting continues to drive behavior even as liking collapses. The present model offers a structural parallel: bondage is an off-surface attractor state maintained by high grasping (g >> 0), in which the restoring gradient toward the surface is dominated by the burden of acquisition. Identity fusion research (Swann et al., 2012) provides an analogous mechanism at the relational level: the fused individual cannot exit a relationship (low Vairagya) regardless of its cost to other dimensions. These are bondage instantiated in specific axes.

The *candidate* qualifier is deliberate throughout this section. The identification of bondage with persistent dūratva is a structural reconstruction, not an established empirical finding. It is put forward as a falsifiable hypothesis: if Proposition 3 is correct, interventions that reduce dūratva should produce the phenomenological signature of increasing freedom, and this signature should distinguish from mere hedonic improvement.

### 7.2 Jivanmukti as stable equilibrium maintenance: proximity vs. tracking

Proposition 4 (candidate model): jivanmukti—liberation while living—is not proximity to the equilibrium surface but the disposition to track it under perturbation. The distinction is the paper’s most portable idea and requires care. A life may reach low dūratva at a given moment by circumstance—fortunate configuration, a windfall of balance. That is proximity, and it is not liberation. Liberation is the tendency of the system to return toward the surface after perturbation—tracking—and to do so continuously as the surface itself deforms under acquisition (Burden Law).

The identification of jivanmukti with tracking must also be situated within the Buddhist liberation literature itself, on three points. First, *anatta* (non-self) (Collins, 1982; Gethin, 1998): the tracking condition is deliberately ownerless—liberation is defined as a disposition of the trajectory, not a property owned by a substantial self. A model that made liberation a stock held by an owner would contradict the tradition it reconstructs; a model that makes it a maintained dynamic is structurally congruent with the anatta doctrine, since what tracks the surface is the configuration, not a proprietor of the configuration. Second, cessation states (*nirodha*) (Gethin, 1998): the geometry does not formalize nirodha, and does not claim to; nirodha is bracketed with the metaphysics of moksha (Limitation 7). What the model can say is that the limiting behavior of the grasping multiplier—inert on the surface, minimal at full release—is the structural shadow, within a livable state space, of what the tradition describes as the stilling of appropriation. Third, the gradual-versus-sudden debate (Gregory, 1987): the geometry is hospitable to both trajectory classes. Gradual liberation is a continuous descent of d(t) with strengthening restoring gradient; sudden liberation is a discontinuous reorganization in which the restoring tendency becomes dominant at once. Both are trajectories in the same state space, which suggests the debate turns on dynamics, not destinations—a reconstruction consonant with the position that the two rhetorics describe one soteriology under different temporal descriptions (Gregory, 1987).

Formally: the model specifies a restoring gradient toward the surface in the Vairagya direction, steeper for larger holdings (Burden Law). Jivanmukti is defined as the stable dominance of this restoring tendency over burden accumulation:

*Jivanmukti condition (tracking criterion)* **jivanmukti iff d(dūratva)/dt < 0 under perturbation for all H > 0**

This is fully consistent with the companion paper’s account of liberation as perpetual self-transcendence (Samanta, 2026): liberation is not a state attained but a process maintained. The geometric form of that process is tracking: the liberated life’s dominant tendency is toward the equilibrium surface, under the continuous perturbation of living in the world. The tracking condition is stated here as the framework for a dynamical model, not as a fully derived dynamical result: the restoring gradient ∇_r d(E) is defined within the static geometry, and the full dynamical model—how d, s, v, r co-evolve under practice and perturbation—is deferred to the subsequent papers of the program (section 14). The condition becomes empirically tractable only once the measurement program of section 12 is complete. A scope constraint merits emphasis here: the geometry aims to capture the *livable structural aspect* of liberation—the trajectory disposition of a life in the world—not metaphysical realization. Aspects of liberation described in the traditions that exceed livable state-space modeling (deep cessation states, *nirodha-samāpatti*, non-dual awareness) remain outside the geometry’s reach by design; this constraint is stated explicitly in Limitation 7 and is not a concession but a declared methodological boundary. The Liberation Coefficient measures the Vairagya dimension of this tracking capacity; the four-axis geometry provides its structural context.

## 8 Buddhist contemplative practice as dimensional rebalancing

*Each canonical path addresses one non-compensable dimension—and why all four are simultaneously required*

**FIGURE 6:**
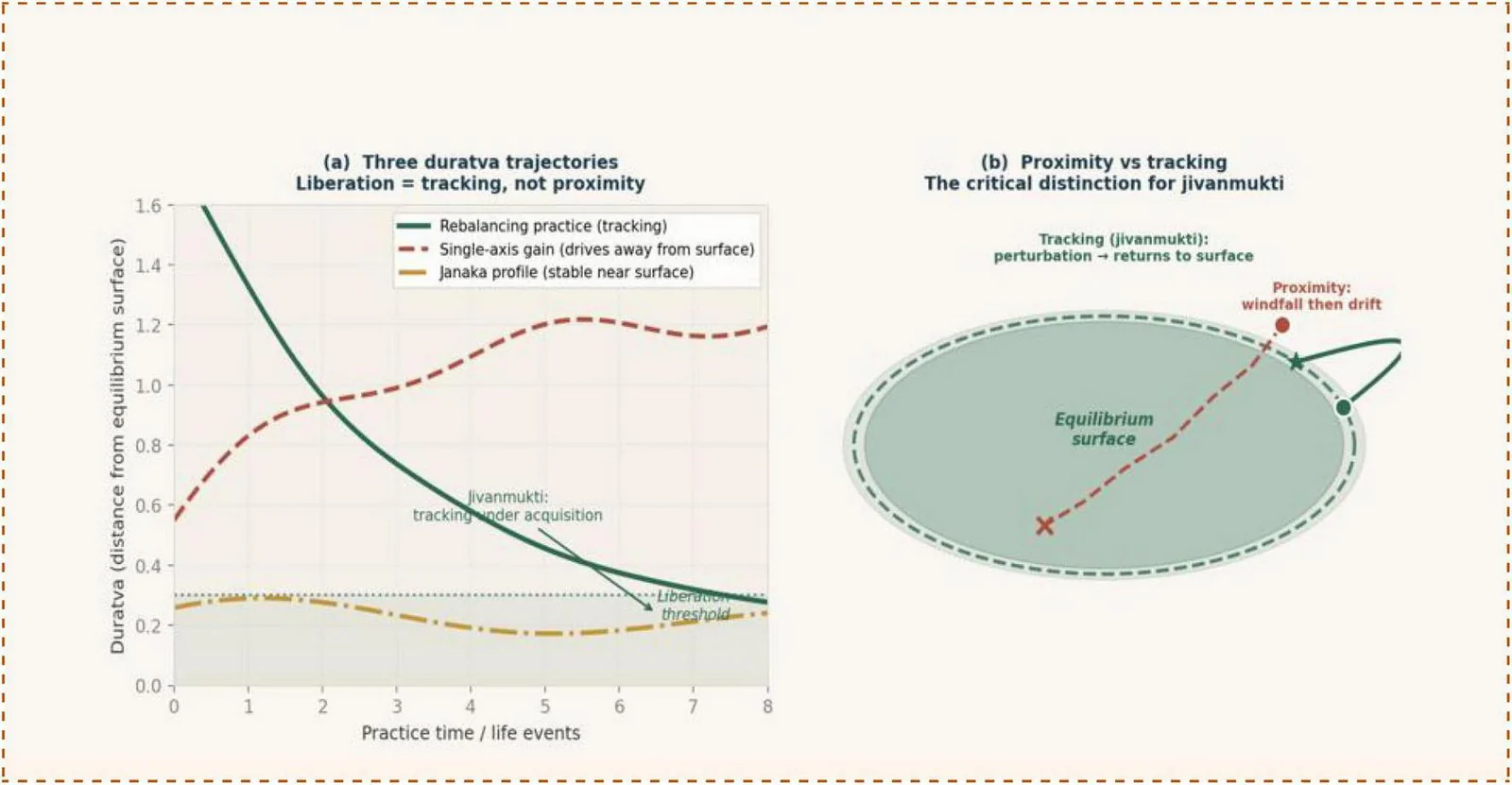
Proximity vs. tracking—the critical distinction for jivanmukti. **(a)** Three dūratva trajectories: rebalancing practice returns the life toward the surface (tracking); single-axis gain drives it away; the Janaka profile maintains stable near-surface position. **(b)** Schematic: proximity is a momentary low dūratva by circumstance; tracking is the disposition to return to the surface under perturbation.

The connection between the equilibrium geometry and Buddhist contemplative practice is structural rather than analogical. Each of the four canonical paths primarily reduces dūratva on one dimension; non-compensability then makes all four simultaneously necessary—not as a doctrinal requirement but as a geometric consequence. Figure 6 maps this in full; the key insight is summarized here before the extended mapping in Supplementary Appendix B.

Karma yoga and seva address the Dhana axis: they ground material engagement above *nyunatā-sīmā* without the accumulation dynamic that, unmodulated by Vairagya, drives the Burden Law. Metta-bhavana and the brahmaviharas address the Relational Reciprocity (S_R) axis (Hofmann et al., 2011): they dissolve the engulfment-isolation duality by cultivating the specific form of connection that affirms rather than consumes—the upekkha (equanimity) component directly touching the Vairagya axis in passing. Vipassana addresses the Vidya axis (Lutz et al., 2008): sustained observation of anicca, dukkha, and anatta makes the fabricated nature of the present orientation visible, which is Vidya in its most precise form. Shamatha and non-attachment cultivation address the Vairagya axis: they develop the baseline stability from which any position can be held and released.

Non-compensability principle for practice: if any one dimension collapses toward zero, dūratva grows toward its maximal value regardless of surplus on the others. All four practices are simultaneously required—this is geometric necessity, not doctrinal prescription.

## 9 Paradigm-case recovery

*The test that distinguishes discovery from stipulation*

**FIGURE 7:**
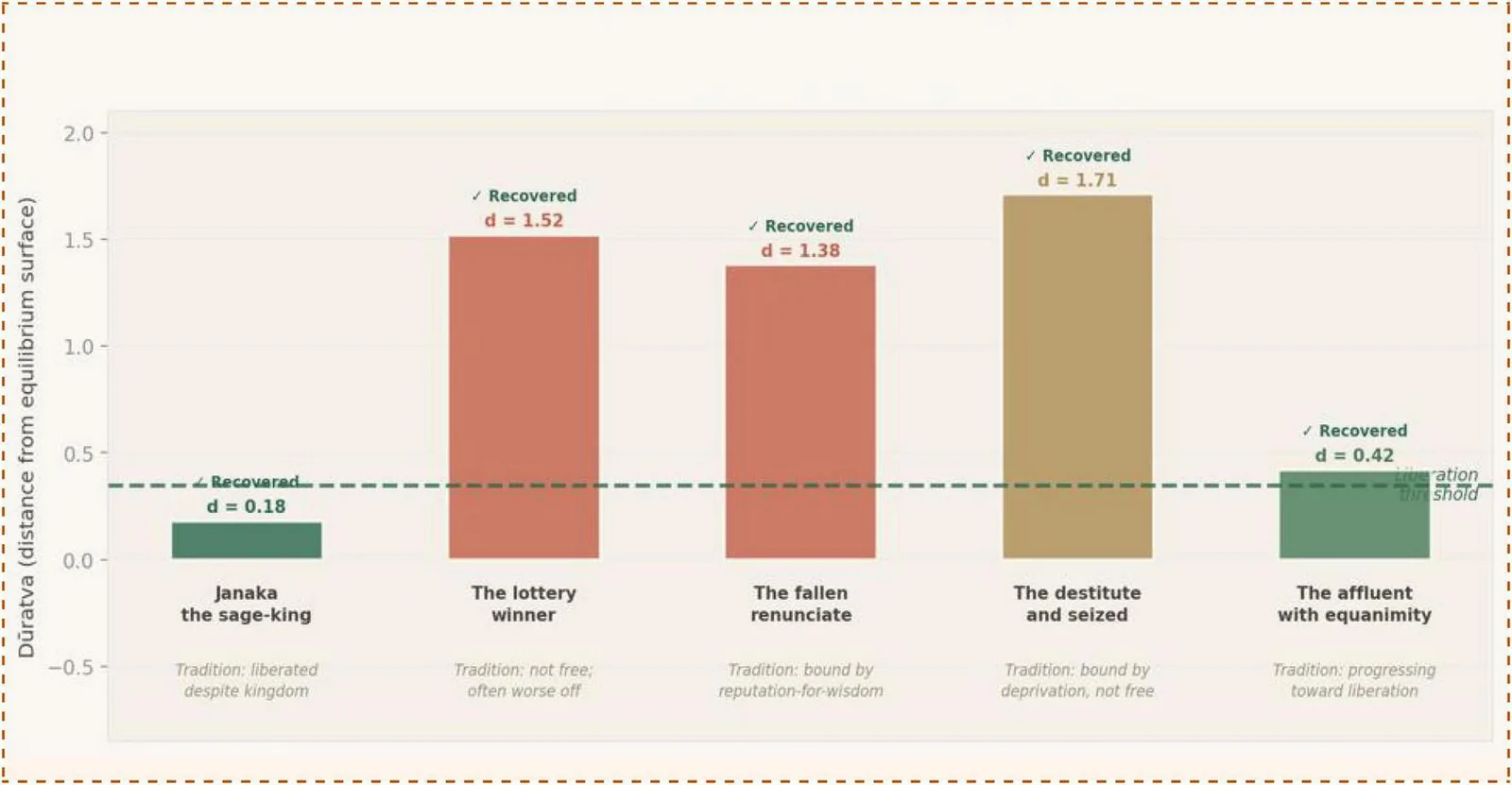
Paradigm-case recovery. The equilibrium geometry reproduces, without parameter tuning, the verdicts that independent traditions established centuries earlier on their own canonical cases. All five recoveries are affirmative, providing non-trivial support that the bondage-dūratva identification is a discovery rather than a definition.

A geometry that explains everything explains nothing. The liberation-bondage identification is a discovery rather than a stipulation only if the model independently reproduces verdicts the tradition established before this framework existed—on cases it never co-ordinated with a manifold. The test is shown in Figure 7.

Five cases are recovered. Janaka: high Dhana, high Vairagya, therefore low grasping, therefore low dūratva—the tradition’s verdict (liberated despite a kingdom) is correct. The lottery winner: sudden high Dhana without proportional Vairagya → grasping high → dūratva far above pre-windfall level—consistent with the empirical literature (Brickman et al., 1978). The fallen renunciate: gain in standing with low Vairagya → far from surface. The destitute-and-seized: low Dhana (the threshold term dominates) and near-zero Vairagya → far from surface for two independent reasons. The contemplative practitioner with equanimity: rising Vairagya matching rising engagement → dūratva decreasing.

## 10 Relation to existing frameworks

*What the equilibrium geometry shares—and where it formally diverges*

**FIGURE 8:**
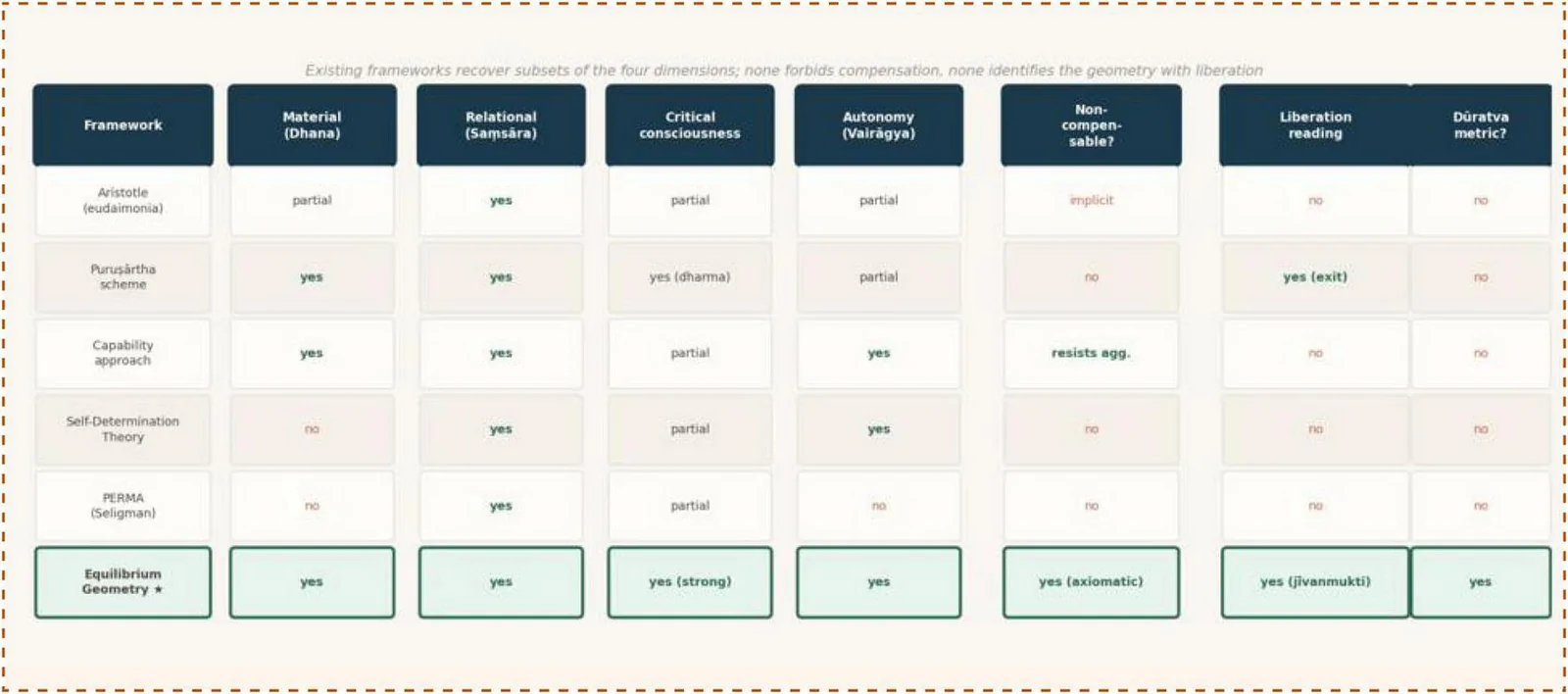
Comparative framework architecture. Existing frameworks recover subsets of the four dimensions; none axiomatically forbids compensation; none provides a dūratva metric; none identifies the geometry with liberation-in-life. The equilibrium geometry (bottom row) makes all three moves simultaneously.

The divergence from existing frameworks runs along one fault line: compensation. Self-determination theory (Deci and Ryan, 2000) recovers three of the four dimensions (Vairagya as autonomy, Samsara as relatedness, a slice of Vidya as competence) but treats them additively; satisfying one need partially offsets unsatisfied others. The capability approach (Sen, 1999; Nussbaum, 2011) is explicitly multidimensional and already resists simple aggregation, but its account of practical reason does not isolate the specific Vidya-capacity to perceive how the conception of the good was installed, and does not provide a distance metric. PERMA (Seligman, 2011) documents five components without forbidding compensation among them or identifying the structure with liberation. The comparison is fully specified in Figure 8.

The framework also speaks directly to a current debate in flourishing science: the relationship between vitality and adaptive wellbeing. Work on subjective vitality (Ryan and Frederick, 1997) has documented that felt energy and aliveness are among the strongest proximal markers of wellbeing, yet vitality can collapse even when composite wellbeing scores—PERMA totals, life satisfaction ratings—remain high. From within additive frameworks, this is anomalous: high total scores should track high wellbeing. The equilibrium geometry offers a structural explanation: vitality collapses when any single dimension approaches its deprivation floor, regardless of surplus elsewhere, because near-threshold shortfall drives the grasping multiplier and compounds dūratva non-linearly. A high-achieving professional with abundant Dhana and S_R but near-zero Vairagya will show collapsing vitality precisely because the grasping coupling on the release axis dominates the total—the burnout-in-success phenomenon (Maslach et al., 2001) that additive models register only as residual variance. Non-compensability thus positions itself at the center of the vitality-and-dynamic-flourishing debate, offering a geometric mechanism where previous accounts have offered only correlational observation.

## 11 Testable hypotheses

*Eight pre-registered predictions and their falsifiers*

The model generates eight testable hypotheses (Table 2). Hypotheses 1 and 2 are partially supported by existing literature; Hypotheses 3–8 are novel. Each is stated as a directional prediction with a specified falsifier. The empirical program required to test these hypotheses is detailed in Supplementary Appendix D. All hypotheses will be submitted to pre-registration prior to data collection; measurement invariance across cultural groups is a pre-registered condition for H1 and H8, not a post-hoc quality check. Pre-registration templates and the dūratva computation script will be made publicly available upon acceptance, in line with open-science best practices.

## 12 Operationalization and measurement

*From theory to instrument: how dūratva becomes computable*

**FIGURE 9:**
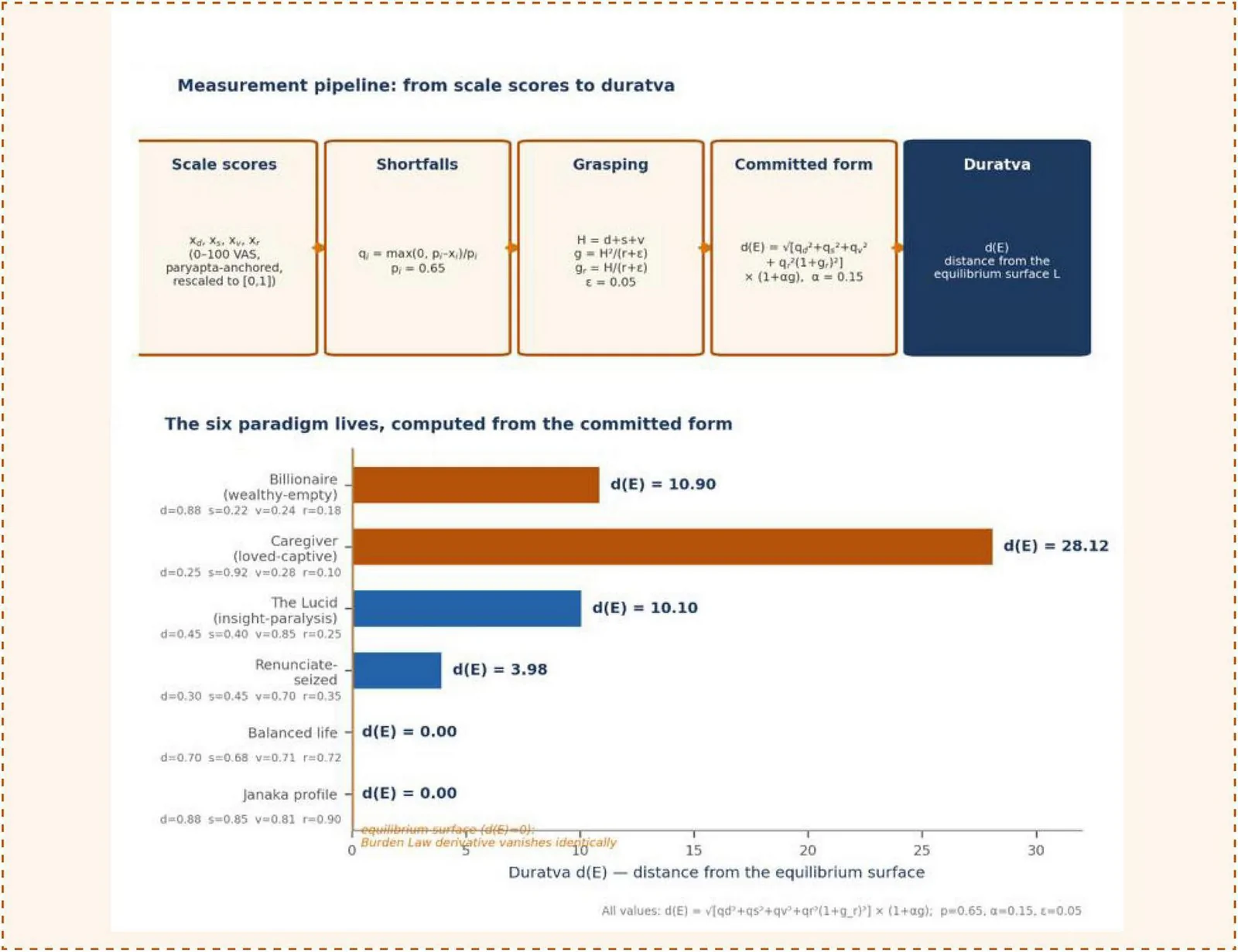
Computing dūratva from scale scores: the measurement pipeline. A worked example showing how four-dimension scale scores are transformed into a dūratva estimate for each of the six paradigm lives. Dūratva is highest for the caregiver (relational over-extension with near-zero Vairagya) and lowest for a balanced profile. The pipeline panel displays a simplified form for readability; all numerical values are computed from the full committed form (Supplementary Appendix A.3) with the parameters stated in Supplementary Appendix B (p_i = 0.65, α = 0.15, ε = 0.05), so figure and appendix use identical parameter values. Symbols are listed in Table 1. **(a)** Measurement pipeline: from scale scores to dūratva. **(b)** The six paradigm lives, computed from the committed form.

Figure 2 maps each dimension to measurable indicators, candidate instruments, and scale anchors. Figure 9 (section 5) shows the computation pipeline from scale scores to dūratva. Supplementary Appendix C provides proposed scale items for each dimension. Three principles govern the measurement program:

**TABLE 1:** Notation.

Symbol	Meaning	Range/value	First defined
E = (d, s, v, r)	Life-state: Dhana, Relational Reciprocity (S_R), Vidya, Vairagya	[0,1]^4^	§5.1
L	Equilibrium surface, locus of balanced states f(E) = 0	subset of [0,1]^4^	§5.1
n_i	Nyunatā-sīmā: deprivation floor for axis i	(0, 0.3]	§5.1/A.2
p_i	Paryāpta: sufficiency anchor for axis i	[0.5, 0.8]	§5.1/A.2
x_i	Observed (normalized) score on axis i	[0, 1]	§5.2
q_i	Normalized shortfall: max(0, p_i - x_i)/p_i	[0, 1]	§5.2/A.3
H	Aggregate holdings: d + s + v	[0, 3]	§6/A.3
g	Global grasping factor: H^2^/(r + ε)	≥0	§5.2/A.3
g_r	Axis-specific grasping pressure: H/(r + ε)	≥0	§5.2/A.3
α	Empirical scaling of the grasping multiplier (candidate 0.15)	>0	A.3
ε	Regularization constant preventing singularity at r = 0 (working value 0.05; empirical parameter)	>0	§5.2/A.3
d(E)	Dūratva: scalar field measuring distance from L	≥0	§5.2/A.3
∇_r d(E)	Restoring gradient in the Vairagya direction	–	§7.2/A.5

All symbols used in the formal sections, with ranges and first definitions.

**TABLE 2:** Eight testable hypotheses with directional predictions and falsifiers.

H	Hypothesis (directional prediction)	Falsifier	Support
H1	Dūratva (balance metric) predicts subjective wellbeing and flourishing above and beyond dimensional totals, across at least three independent samples.	Dimensional totals consistently outpredict dūratva in regression models across diverse samples (*n* > 200 per study, *p* < 0.05).	Partial (Brickman et al., 1978; Easterlin, 1974; Kahneman and Deaton, 2010)
H2	Unmatched single-axis gains (top-tercile gain on one dimension, no gain on others) reduce long-run flourishing at 12-month follow-up relative to balanced-gain controls.	Single-axis gains reliably maintain or increase flourishing outcomes at 12-month follow-up.	Partial (Brickman et al., 1978; Kahneman and Deaton, 2010)
H3	High Vidya with low Vairagya (upper tertile Vidya, lower tertile Vairagya) predicts a specific distress profile—insight-paralysis—distinct from both low-Vidya and low-Vairagya profiles on a cluster analysis. Cluster number fixed a priori at *k* = 4 (the four quadrants of the Vidya–Vairagya plane); silhouette scores and the gap statistic serve as pre-registered robustness checks, not selection criteria.	Insight-paralysis cluster does not emerge; high-Vidya/low-Vairagya profile is indistinguishable from low-Vidya profiles.	Novel
H4	Recovery rate after a matched life perturbation (bereavement, job loss) scales with Vairagya at baseline—measured by LC (Samanta, 2026) or the proposed Vairagya Scale—after controlling for perturbation severity.	Recovery rate is independent of baseline Vairagya and LC in multivariate regression (beta < 0.10).	Indirect (Deci and Ryan, 2000; Samanta, 2026)
H5	Among practitioners above all four sufficiency thresholds, an 8-week rebalancing intervention (addressing depleted dimensions) outperforms single-axis intensification on composite dūratva reduction at 3-month follow-up.	Single-axis intensification and rebalancing show equivalent dūratva outcomes in pre-registered RCT.	Novel
H6	Independent coders, blind to the model, sort cross-tradition symbolic elements (paradigm cases from Buddhist, Vedantic, Sufi, and Christian traditions) onto the four dimensions above chance level (chi-squared test, *p* < 0.01, expected > 25%). Stimulus set selected and frozen under pre-registered criteria before rater recruitment (D.6), with equal representation across dimensions to preserve the 25% chance baseline.	Cross-tradition sorting accuracy is at chance (25%) or below in pre-registered rater study.	Novel (rater-recovery test)
H7	Buddhist practitioners whose practice simultaneously addresses three or more dimensions show steeper LC growth trajectories over 6 months than single-practice controls, using the LC measurement protocol of Samanta (2026).	LC growth rates are equivalent across single-practice and multi-practice groups in controlled longitudinal study.	Predicted from (Samanta, 2026)
H8	PERMA subscale gains cluster dimensionally by practice type: shamatha practitioners show stronger E+M (Engagement+Meaning) gains; metta practitioners show stronger R+P (Relationships+Positive emotion) gains—after controlling for total practice hours.	PERMA gains are uniformly distributed across subscales regardless of practice type (F-ratio < 2.0).	Partially (Lally et al., 2010)

“Novel” indicates no prior direct test. “Partial” for H1 and H2 denotes indirect support from the hedonic-adaptation and income–wellbeing literatures; the dūratva measure itself has not yet been operationalized or directly tested in any published study. Full operationalization and study designs are in Supplementary Appendix D.

First, scales should be anchored to *paryāpta* (enough) rather than to cultural maxima. Standard wellbeing scales typically ask “how satisfied are you with your financial situation?” on a scale from very dissatisfied to very satisfied, which anchors at a culturally-variable maximum. The proposed Dhana Sufficiency Scale anchors at *paryāpta*: “Does your material situation provide a stable margin between your resources and your needs—not maximum wealth, but enough?” This anchor change is conceptually consequential and requires new psychometric development.

Second, Vairagya measurement already has a published anchor: the Liberation Coefficient (LC) of Samanta, (2026) directly measures position-fixation approaching zero, which is the behavioral signature of high Vairagya. This provides an externally validated anchor for the fourth dimension that is absent from the other three.

Third, Vidya requires genuinely new scale development. No existing validated instrument captures the specific construct of perceiving how one’s conception of the good was constructed and installed. The Critical Consciousness Scale (Diemer et al., 2017) addresses the political form of this capacity; a contemplative-context adaptation is needed. Supplementary Appendix C provides twelve candidate items.

## 13 Limitations

*Seven places where the framework could be wrong*

1. The framework is theoretical, not yet validated. The phenomenological composites (the four people of the Introduction) are *anubhava*—heuristic illustrations that motivate the structure—not *pramana* (evidence that confirms it). All eight hypotheses remain to be tested against data.

2. The committed form of f is one candidate among possible forms with the correct qualitative behavior (steep bounded growth as any axis approaches its threshold). Alternative mathematical forms—non-Euclidean distance metrics, Riemannian manifolds incorporating inter-axis curvature, Copula-based dependence structures—may provide better fits to data when measurement instruments are developed.

3. The non-compensability axiom is falsifiable (H2). Demonstrated compensability—empirical wellbeing data showing that surplus on one dimension reliably offsets collapse on another—would break the central geometry. This is stated not as a caveat but as the framework’s defining risk.

3a. Not all hypotheses bear equally on the core structure. It is worth distinguishing those whose failure would require revision of the fundamental geometry from those whose failure would require only parameter adjustment or boundary-condition revision.

*Core-threatening* (failure would require revision of the non-compensability axiom or dimensional structure): H2—if single-axis gains reliably maintain long-run flourishing, the non-compensability axiom would require revision; H3—if high-Vidya/low-Vairagya profiles are indistinguishable from low-Vidya profiles, the structural independence of Vidya and Vairagya would be disconfirmed; H6—if raters cannot sort cross-tradition symbols above chance, the claim that exactly four dimensions are structurally encoded in contemplative traditions would be weakened (though the geometry could remain intact with a different cardinality).

*Ancillary* (failure would require parameter revision or boundary-condition amendment, but leaves the non-compensable geometry intact): H1—failure would indicate that the current operationalization of dūratva lacks predictive validity, not that the geometry is wrong; H4, H5, H7, H8—failure would constrain the scope of the framework’s practical claims without undermining the structural axiom.

This distinction is the framework’s Lakatosian architecture: a hard core (non-compensability, functional irreducibility of the four dimensions) and a protective belt (parameter values, operationalization choices, boundary conditions). The hard core can be defended by revising the belt; but H2, H3, and H6 strike at the core directly.

4. The cardinality of the dimensions (exactly four) is a structural claim that can be weakened by cross-cultural evidence. A well-developed tradition that encodes a genuinely different dimensional structure of the good life—not a relabeling but a different cardinality—would require extending or revising the model. The rater-recovery test (H6) provides the empirical avenue for this challenge. This limitation deserves foregrounding rather than burial: the four dimensions draw heavily on Sanskrit Buddhist concepts, and the paryāpta anchor in particular must translate across cultural contexts in which sufficiency norms vary substantially. The proposed translation strategy is functional rather than absolute: paryāpta is anchored to the margin between resources and needs—a relational quantity—rather than to any absolute level of holdings, which is what allows the anchor to travel across economies and cultures where the same absolute holdings mean different things. Measurement invariance testing (multi-group configural, metric, and scalar CFA across at least two culturally distinct samples) is built into the measurement program (Supplementary Appendix D, Study 3) as the empirical check on whether this strategy succeeds.

5. The use of *Samsara* and *Vairagya* as technical terms with restricted meanings differs from their usage in Buddhist studies. Specialists may resist the terminological appropriation even when the paper explains its rationale. The alternative labels—Relational Reciprocity (S), Autonomy-as-Non-Seizure (V)—are used in all operationalization contexts to minimize this resistance.

6. The iconographic encoding claim (that the four dimensions are structurally preserved in certain classical symbolic systems) is offered as an independently evaluable hypothesis (H6), not as evidence for the model. The rater-recovery test must precede any appeal to iconographic convergence as support.

7. The framework reconstructs *jivanmukti* (liberation while living) and explicitly brackets *moksha* entire—the metaphysical dimensions of liberation, liberation-at-death (*videhamukti*), and non-dual metaphysics. The traditions mean more by liberation than this model formalizes. The model takes the livable structure and acknowledges the remainder by declared method.

## 14 Position of the present paper within the research program

The present paper develops the theoretical foundation for a broader program of inquiry into the relationship between Buddhist contemplative practice and psychological flourishing. Its primary aim is structural rather than mechanistic. Specifically, it proposes that flourishing can be understood as a four-dimensional equilibrium composed of sustaining sufficiency (Dhana), relational reciprocity (Samsara), critical consciousness (Vidya), and autonomy-as-non-seizure (Vairagya), and that these dimensions are non-compensable. The central contribution of the paper is therefore the formalization of flourishing as a geometric problem and the introduction of dūratva as a measure of distance from equilibrium.

Several important questions are intentionally left open. The present paper does not attempt to explain the neurophysiological mechanisms through which contemplative practices alter an individual’s position within this geometry. Nor does it seek to establish how the proposed dimensions are embodied in movement, ritual, posture, or other somatic forms of practice. Likewise, questions concerning intervention design, educational implementation, clinical translation, and the ethical implications of integrating Buddhist practices into contemporary positive psychology are beyond the scope of the current article.

These questions are addressed in the subsequent papers of the program. The second paper examines the neurophysiological processes through which repeated contemplative practice becomes consolidated as stable behavioral and psychological change. The third paper investigates the embodied and somatic dimensions of contemplative formation, exploring how movement and ritual may function as technologies of psychological cultivation. The fourth paper translates the framework into clinical and educational settings, proposing practical principles for the design of evidence-informed contemplative interventions. The fifth paper expands the discussion cross-culturally and ethically, examining whether the proposed principles reflect universal features of human development and how Buddhist practices may be integrated into positive psychology without losing their original liberatory orientation.

Taken together, the five papers are intended to form a coherent research program. The present article establishes the geometry. The subsequent articles explore the mechanisms, embodiments, applications, and broader implications of that geometry. Whether the framework ultimately succeeds will depend not on the elegance of the theory alone, but on the empirical and practical investigations that follow from it.

The instruments developed in Supplementary Appendix D, the computation script for dūratva, and the pre-registration templates for H1–H8 will be made publicly available upon acceptance. Research teams working in positive psychology, contemplative science, clinical psychology, cross-cultural wellbeing, and Buddhist studies are explicitly invited to adopt, adapt, challenge, and extend these protocols. The framework does not depend on a single group’s work—it depends on independent empirical scrutiny across diverse settings, populations, and traditions. A theory of human flourishing that is not tested by many hands is not yet a theory of human flourishing; it is a proposal waiting for the world to answer it.

## 15 Conclusion

The four people of the Introduction are not reporting insufficient quantity. They are reporting structural imbalance—a gap between the configuration of their lives and the balance that flourishing requires. The equilibrium geometry formalizes what those reports have always been saying: suffering is distance from one’s own balance, not distance from someone else’s maximum.

The paper’s contribution to the Research Topic is twofold. Theoretically, it proposes non-compensability as the missing structural axiom that converts multidimensional wellbeing science into a framework capable of explaining the surplus-with-suffering phenomenon. Practically, it shows why all four canonical Buddhist paths are simultaneously required: each path addresses one non-compensable dimension, and the geometry forbids any of them to substitute for the others.

Whether the framework earns the name of a theory will depend on whether the committed form of the metric, the non-compensability axiom, and the identification of jivanmukti with tracking survive the tests proposed in section 11. These are the framework’s honest risks, and they are the same as saying: this is a framework that can be wrong.

*Flourishing is equilibrium seen from within. Liberation is equilibrium seen from the standing-back. Dūratva measures both.*

## Data Availability

The original contributions presented in the study are included in the article/Supplementary material, further inquiries can be directed to the corresponding author.
