# Supplementary material for: Why balance matters more than totals: a candidate structural model of non-compensable wellbeing, duratva, and Buddhist contemplative practice
Source: Front Psychol. 2026 Aug 31;17:1902817. doi: 10.3389/fpsyg.2026.1902817 (PMC13572624; doi:10.3389/fpsyg.2026.1902817)
Supplement: Supplementary file 1 [file Supplementary_file_1.pdf]

Appendix A: Mathematical Derivations

A.1 State Space and Notation

Let $E = (d, s, v, r) \in [0,1]^4$ denote a life-state, with coordinates:

$d \in [0,1]$ — Dhana (sustaining sufficiency)

$s \in [0,1]$ — Samsara (relational reciprocity)

$v \in [0,1]$ — Vidya (critical consciousness)

$r \in [0,1]$ — Vairagya (autonomy-as-non-seizure)

A.2 Threshold Parameters

Each axis has two threshold parameters:

$n_i \in (0, 0.3]$ — nyunatā-sīmā: deprivation floor for dimension i

$p_i \in [0.5, 0.8]$ — paryapta: sufficiency anchor for dimension i

$\varepsilon > 0$ — regularization constant preventing singularity at $r = 0$ (working value 0.05); treated as an empirical parameter, with sensitivity analysis reported in Appendix B

These are empirical parameters. The structural claim is that both exist and have distinct effects, not their precise values.

A.3 Shortfall, Grasping, and Duratva

Normalised shortfall on axis i ($i \in \{d,s,v,r\}$) $q_i = \max(0, p_i - x_i) / p_i$

Aggregate holdings $H = d + s + v$ **(three holding-axes; r is the release axis)**

Grasping (derived quantity — NOT a fifth coordinate) $g = H^2 / (r + \varepsilon)$ **where** $\varepsilon = 0.05$

Duratva — committed candidate form $d(E) = \mathrm{sqrt}[q_d^2 + q_s^2 + q_v^2 + q_r^2(1 + g_r)^2] \times (1 + \alpha \cdot g)$

where $g_r = H / (r + \varepsilon)$ is the axis-specific grasping pressure on Vairagya, and α is an empirical scaling parameter (candidate value: $\alpha = 0.15$).

This form was chosen as the first test case for three reasons: (i) it admits closed-form parameter estimation with interpretable coefficients; (ii) each term has direct conceptual meaning — the inner square root measures unmet need, the outer multiplier measures the compounding cost of holding; and (iii) the three structural properties below are analytically transparent without numerical fitting. Whether this form or an alternative (Riemannian, Copula-based) provides the best empirical fit is precisely what H1–H5 are designed to determine (Limitation 2).

Three qualitative properties of d(E) — the conceptual handle on the committed form:

(a) Non-compensability. As any $q_i \to 1$ (a dimension collapses toward its deprivation floor), d(E) grows steeply toward its maximal finite bound regardless of surplus on the remaining axes. The normalisation $q_i \in [0,1]$ excludes true mathematical divergence; the steep bounded growth is sufficient to dominate all compensating surplus, which is the operative content of the non-compensability claim.

(b) Grasping-mediated amplification of Vairagya shortfall. The Vairagya shortfall enters as $q_r^2(1 + g_r)^2$, so near-zero Vairagya under high holdings amplifies d(E) quadratically through the axis-specific grasping pressure. Here lies the geometric mechanism behind the Burden Law: holding more, with low Vairagya, is structurally costly.

(c) Euclidean reduction. When $\alpha \to 0$ or holdings $\to 0$ (pure release or empty holding), $g \to 0$, $g_r \to 0$, and d(E) reduces to the Euclidean shortfall distance $\sqrt{[q_d^2 + q_s^2 + q_v^2 + q_r^2]}$. This limit confirms that the grasping structure is an additional layer on top of a baseline distance — it enriches without replacing.

Terminology: d(E) is a scalar field (a point-to-surface distance), not a metric in the strict sense; metric axioms are defined for pairwise distances and do not apply here.

A.4 Burden Law — Formal Derivation (complete chain rule)

Write $d(E) = S \times M$, with $S = \mathrm{sqrt}[q_d^2 + q_s^2 + q_v^2 + q_r^2(1 + g_r)^2]$ the inner square root and $M = 1 + \alpha g$ the grasping multiplier. Consider a gain dH on the holding axes (d, s, v), with the shortfall profile and r held fixed. The gain acts through both factors, since $\partial g/\partial H = 2H/(r + \varepsilon)$ and $\partial g_r/\partial H = 1/(r + \varepsilon)$. The complete chain rule therefore yields two terms — the multiplier (global-grasping) term and the inner-coupling (axis-specific) term:

Burden Law — marginal cost of acquisition (full form) $\partial d(E)/\partial H = S \times \alpha \times 2H/(r+\varepsilon) + M \times q\_r^2(1+g\_r) / [(r+\varepsilon) \times S] = (1/(r+\varepsilon)) \times [2\alpha H\cdot S + M\cdot q\_r^2(1+g\_r)/S]$

Both terms are non-negative, and their structure yields three exact results. (a) The derivative is strictly positive for every life off the equilibrium surface ($S > 0$): wherever any shortfall remains, acquisition increases duratva. (b) The derivative vanishes identically on the surface: as all $q\_i \to 0$, $S \to 0$ and $q\_r^2(1+g\_r)/S \to 0$, so both terms go to zero — acquisition adds no duratva to a fully balanced life. That is the precise content of the Janaka condition: residence on the equilibrium surface with Vairagya at the level of the holdings. Note that the algebraic identity $r = H$ is not generally attainable within $[0,1]^4$ when $H > 1$, so the condition must be stated in terms of the surface, not that identity. (c) The derivative is strictly decreasing in r, through the $1/(r + \varepsilon)$ prefactor and through g_r: among lives with the same holdings and shortfall profile, higher Vairagya strictly minimises the burden rate. Both terms enter all numerical work reported in Appendix B; omitting the inner-coupling term $M\cdot q\_r^2(1+g\_r)/[(r+\varepsilon)\cdot S]$ would understate the marginal cost of acquisition at low Vairagya.

A.5 Tracking Condition (Jivanmukti)

Define the restoring gradient as $\nabla\_r\, d(E)$ — the rate at which duratva decreases as Vairagya increases. Jivanmukti is the stable dominance of this restoring tendency over the Burden Law's acquisition-driven increase:

Jivanmukti (dynamic tracking condition) $L(t) = \text{True}$ **iff** $|\nabla\_r\, d(E)| > |\partial d(E)/\partial H \times \Delta H/\Delta t|$ **for all perturbations** ΔH

This condition states that the Vairagya-driven return force exceeds the burden force at the current rate of acquisition. It is an empirical condition — whether a given life satisfies it is a question about the practitioner's actual Vairagya and the rate of new acquisition — and it connects directly to the Liberation Coefficient of Samanta [9]: $LC \to \infty$ corresponds to the tracking condition being robustly satisfied. The condition is stated as the framework for a dynamical model rather than as a derived dynamical result: the restoring gradient is defined within the static geometry, and the full co-evolution of (d, s, v, r) under practice and perturbation is deferred to subsequent papers of the programme. The condition becomes empirically tractable once the measurement programme (Appendix D) is complete.

Appendix B: Worked Numerical Examples

The following six examples use hypothetical scale scores to illustrate duratva computation and to demonstrate the Burden Law. All values are computed from the full committed form of A.3 — $d(E) = \sqrt{q_d^2 + q_s^2 + q_v^2 + q_r^2(1 + g_r)^2} \times (1 + \alpha \cdot g)$ — by a single computation script with identical parameters throughout: $p_i = 0.65$ (paryapta), $n_i = 0.20$ (nyunatasima), $\alpha = 0.15$, $\varepsilon = 0.05$. Figure 4 is generated by that same script, so the figure and this appendix are numerically identical. Because duratva is a relative measure of distance from equilibrium — not an absolute scale with privileged units — the magnitudes matter only through their ordering and ratios, both of which are the model's actual commitments. Cases B.1, B.2, B.5, and B.6 are the four figures of the Introduction, now computed.

B.1 The Billionaire — high Dhana, low others (the wealthy-empty)

**Scores**	**d=0.88 s=0.22 v=0.24 r=0.18**
**Shortfalls q_i**	$q_d=0.00$ $q_s=0.66$ $q_v=0.63$ $q_r=0.72$
**H, g, g_r**	$H=1.34$ $g=1.34^2/0.23=7.81$ $g_r=1.34/0.23=5.83$
**d(E)**	**$d(E) = \sqrt{0 + 0.44 + 0.40 + 0.523\times(1+5.83)^2} \times (1+0.15\times7.81) = 5.02 \times 2.17 \approx 10.90$**
**Burden rate ∂d/∂H**	**≈ 15.5 — steep: every further acquisition is expensive**
**Interpretation**	*Very high duratva. The single Dhana surplus provides zero reduction; the grasping coupling on near-collapsed Vairagya dominates.*

B.2 The Caregiver — maximal S_R, near-zero Vairagya (the loved-captive)

**Scores**	**d=0.25 s=0.92 v=0.28 r=0.10**
**Shortfalls q_i**	$q_d=0.62$ $q_s=0.00$ $q_v=0.57$ $q_r=0.85$
**H, g, g_r**	$H=1.45$ $g=1.45^2/0.15=14.02$ $g_r=1.45/0.15=9.67$
**d(E)**	**$d(E) = \sqrt{0.38 + 0 + 0.32 + 0.716\times(1+9.67)^2} \times (1+0.15\times14.02) = 9.06 \times 3.10 \approx 28.12$**
**Burden rate ∂d/∂H**	**≈ 43.7 — the steepest of the six: further relational holdings are heaviest of all**
**Interpretation**	*Highest duratva of all cases. Relational maximum with near-zero Vairagya produces extreme burden — over-connection cannot compensate for the collapsed release axis.*

B.3 Balanced life — all four axes near paryapta

Scores	d=0.70 s=0.68 v=0.71 r=0.72
**Shortfalls q_i**	q_d=0.00 q_s=0.00 q_v=0.00 q_r=0.00
**H, g, g_r**	H=2.09 $g=2.09^2/0.77=5.67$ g_r=2.71
**d(E)**	**d(E) = sqrt[0] × (1+0.15×5.67) = 0.00**
**Burden rate ∂d/∂H**	**= 0 exactly — on the surface, acquisition is weightless**
**Interpretation**	*Zero duratva. All axes at or above paryapta; the grasping multiplier is inert on a zero base, and the Burden Law derivative vanishes identically (A.4, result b).*

B.4 Janaka profile — high all-round, especially Vairagya

Scores	d=0.88 s=0.85 v=0.81 r=0.90
**Shortfalls q_i**	q_d=0.00 q_s=0.00 q_v=0.00 q_r=0.00
**H, g, g_r**	H=2.54 $g=2.54^2/0.95=6.79$ g_r=2.67
**d(E)**	**d(E) = sqrt[0] × (1+0.15×6.79) = 0.00**
**Burden rate ∂d/∂H**	**= 0 exactly — the kingdom is held weightlessly**
**Interpretation**	*Zero duratva despite vast holdings. The Janaka condition in its exact form: residence on the equilibrium surface with Vairagya at the level of the holdings. Acquisition adds nothing because there is no shortfall for grasping to couple to.*

B.5 The Lucid — high Vidya, low Vairagya (insight-paralysis)

Scores	d=0.45 s=0.40 v=0.85 r=0.25
**Shortfalls q_i**	q_d=0.31 q_s=0.38 q_v=0.00 q_r=0.62
**H, g, g_r**	H=1.70 $g=1.70^2/0.30=9.63$ g_r=1.70/0.30=5.67
**d(E)**	**d(E) = $\mathrm{sqrt}[0.09 + 0.15 + 0 + 0.379\times(1+5.67)^2] \times (1+0.15\times9.63) = 4.13 \times 2.44 \approx 10.10$**
**Burden rate ∂d/∂H**	**≈ 12.0**
**Interpretation**	*The seer-without-release (Section 4). Vidya at surplus buys no reduction: duratva is comparable to the Billionaire's, though the surplus axis is insight rather than wealth — clarity has become its own captivity. This is the H3 profile.*

B.6 The Renunciate-Seized — renunciation held as identity

Scores	d=0.30 s=0.45 v=0.70 r=0.35
**Shortfalls q_i**	q_d=0.54 q_s=0.31 q_v=0.00 q_r=0.46
**H, g, g_r**	H=1.45 g=1.45^2/0.40=5.26 g_r=1.45/0.40=3.62
**d(E)**	**d(E) = sqrt[0.29 + 0.09 + 0 + 0.213×$(1+3.62)^2$] × (1+0.15×5.26) = 2.22 × 1.79 ≈ 3.98**
**Burden rate ∂d/∂H**	**≈ 4.4**
**Interpretation**	***"Free from the world, not free from the person who is free from the world." Moderate Vairagya seized around the renunciate identity itself, with Dhana pressed toward its floor: duratva well above the surface, though far below the unbalanced-surplus cases. Renunciation without release is a position, not a liberation.***

B.7 Sensitivity of results to the regularization constant ε

Because ε enters the denominators of g and g_r, its influence is largest where r is small. Recomputing the two low-r cases across ε ∈ {0.01, 0.05, 0.10}: the Caregiver yields d(E) = 46.5, 28.1, 18.1 and the Billionaire yields d(E) = 14.3, 10.9, 8.4 respectively. The quantitative values shift, as expected for a regularization parameter; the ordering of all six cases (Caregiver > Billionaire > Lucid > Renunciate-Seized > Balanced = Janaka = 0) is invariant across the range, as are all qualitative conclusions. That is the operational sense in which ε is an empirical parameter to be estimated rather than a theoretical commitment: it scales the low-r regime without reordering it. The clinical ordering of profiles — and therefore all directional predictions of H1–H8 — is robust to the choice of regularization value within a plausible empirical range; only the absolute magnitude of duratva in low-Vairagya regimes is sensitive to ε, and that sensitivity is itself informative about the degree of grasping amplification in the Caregiver profile.

Appendix C: Proposed Scale Items (Draft — Pre-Validation)

The following items are proposed as a starting point for instrument development. They are not validated; psychometric development and pre-registration are required before research use. All items use a 0–100 visual analogue scale anchored to paryapta (enough) rather than to a maximum. Items marked [R] are reverse-coded. Within the Vidya block, items are additionally

tagged [P] (perceptual sub-capacity: perceiving that one's desires were socially constructed) or [E] (evaluative sub-capacity: critically assessing whether those desires are worth endorsing), following the bifurcation of Section 2.3; the separability of the two sub-factors is a pre-registered target of Study 2 (Appendix D).

Dhana (D — Sustaining Sufficiency)

D1. My material situation provides a stable margin between my resources and my needs, freeing my attention for what matters.

D2. I can meet my essential needs without chronic anxiety about resources.

D3. I have enough — not maximum, but a stable enough — to engage fully with my life.

D4. [R] I am preoccupied with securing resources even when my basic needs are met.

D5. My material situation feels like a foundation rather than a weight.

Samsara (S — Relational Reciprocity)

S1. My connections with others feel like they affirm rather than consume me.

S2. I can be fully present in my relationships without losing myself in them.

S3. My relational life involves genuine reciprocity — I give and I receive.

S4. [R] I feel either isolated (too little connection) or engulfed (too much obligation).

S5. My relationships leave me feeling more myself, not less.

Vidya (V — Critical Consciousness)

V1. [P] I can identify which of my desires and values were shaped by the social context I grew up in, rather than freely chosen.

V2. [E] When I pursue a goal, I can examine whether that goal truly reflects what I value or whether it was installed by others' expectations.

V3. [P] I notice when I am acting from absorbed social norms rather than from deliberate values.

V4. [R][P] My conceptions of success, worth, and the good life feel simply natural to me — I rarely question where they came from.

V5. [P] I am able to perceive the constructed nature of my own preferences without this perception paralyzing me.

V6. [E] When I recognise that a desire of mine was installed by others, I can still ask — and answer — whether it is a desire worth keeping.

V7. [R][E] Even when I see where a value of mine came from, I have no way of deciding whether to keep it.

Vairagya (R — Autonomy-as-Non-Seizure)

R1. I have the structural freedom to exit roles, relationships, or resource configurations that no longer serve my deepest values, without catastrophic loss.

R2. I can decline requests, offers, or opportunities — even attractive ones — without overwhelming anxiety.

R3. I am not seized by what I hold: my holdings do not grip me more than I grip them.

R4. [R] I feel unable to exit my current situation even when it is no longer serving me.

R5. My sense of self is not dependent on maintaining any particular role, position, or achievement.

R6. [Validated anchor] Liberation Coefficient score (LC) from Samanta [2] behavioural battery. (Cross-validate against R1-R5.)

Appendix D: Empirical Research Programme — Proposed Study Designs

The following eight studies constitute the minimum empirical programme required to adequately test the framework. They are presented in logical rather than temporal order; Studies 1–3 must precede the others.

D.1 Study 1: Scale development

Develop and refine the four-dimension instrument using mixed-methods approach. Cognitive interviewing (n=20), content validity (n=10 expert raters), and pilot testing (n=150). Target: four subscales with internal consistency $\alpha > 0.80$ and test-retest reliability ICC > 0.75.

D.2 Study 2: Exploratory factor analysis

EFA on the four-dimension scale (n=300 minimum). Pre-registered prediction: four-factor structure with minimal cross-loadings (<0.30). The EFA will additionally test whether the Vidya [P] and [E] items load on separable, correlated sub-factors within the Vidya block (Section 2.3). Secondary outcome: comparison of duratva (distance from balance) vs PERMA composite as predictors of validated wellbeing outcomes.

D.3 Study 3: Confirmatory factor analysis and convergent validity

CFA (n=500) confirming four-factor structure. Convergent validity with PERMA-H, BWAS, BPNS, and LC [2] scale. Duratva prediction of SWLS, PANAS, and GHQ-12 above and beyond PERMA total (H1). Multi-group CFA will test measurement invariance (configural, metric, scalar) across at least two culturally distinct samples, as the empirical check on the paryapta translation strategy (Limitation 4). The H3 insight-paralysis cluster analysis will be pre-registered on these data with k = 4 fixed a priori — the four quadrants of the Vidya–Vairagya plane — with silhouette scores and the gap statistic as robustness checks, not selection criteria.

D.4 Study 4: Longitudinal burden law validation

Prospective cohort (n=200, 12-month follow-up). Measure baseline duratva; track single-axis gainers (occupational success without relational/Vairagya development) vs balanced gainers. Primary outcome: duratva trajectory and 12-month wellbeing. Tests H2.

D.5 Study 5: Rebalancing RCT

Pre-registered RCT (n=120): rebalancing intervention (8 weeks, addressing two most-depleted dimensions) vs matched-intensity single-axis intensification. Outcome: duratva at 3 months. Tests H5.

D.6 Study 6: Cross-tradition rater-recovery study

Blind sorting study. 40 independent raters assign 80 symbolic elements (20 per tradition: Buddhist, Vedantic, Sufi, Christian) to four dimensions without model knowledge. Stimulus pre-registration: the 80 elements are selected before rater recruitment by two independent tradition experts who are blind to the model, under frozen written criteria (canonical status within the source tradition; symbolic rather than doctrinal-propositional character; balanced representation, 20 per dimension across the full set, to preserve the 25% chance baseline). The selection list and criteria are pre-registered together with the prediction; no element may be added, removed, or re-described after registration. Pre-registered criterion: accuracy > 35% per dimension (vs 25% chance). Chi-squared test. Tests H6.

D.7 Study 7: Buddhist practitioner trajectory study

Longitudinal cohort (n=100, 6-month follow-up). Classify practitioners by primary practice type (single-path vs multi-path). Measure LC [2] and duratva monthly. Prediction: multi-path practitioners show steeper LC growth rate. Tests H7.

D.8 Study 8: Perturbation and recovery study

Prospective matched cohort: individuals experiencing a major life perturbation (bereavement, job loss) assessed at 1 month and 6 months post-event. Baseline Vairagya (LC [2] + Vairagya Scale) as predictor of recovery rate. Tests H4.
